# Supplementary material for: iHeard STL: Development and first year findings from a local surveillance and rapid response system for addressing COVID-19 and other health misinformation
Source: PLoS One. 2023 Nov 3;18(11):e0293288. doi: 10.1371/journal.pone.0293288 (PMC10624282; doi:10.1371/journal.pone.0293288)
Supplement: S1 Table — (DOCX) [file pone.0293288.s001.docx]

**Table S1.** Exposure and belief definitions, measures, and use.

| **Construct** | **Measure** | **Definition and Statistic** | **Example Use** |
| --- | --- | --- | --- |
| Exposure | First exposure | Definition: binary (yes/no) variable where panel members who responded yes to having heard/read/seen the information item in the 7 days prior to the first time they ever answered the question regarding the information item were coded as a yes. | Sub-group analyses |
|  | Weekly exposure | Definition: percentage of panel members who responded yes to having heard/read/seen the information item in the 7 days prior to a given week    (Panel members who reported being exposed to the information item in the given week/panel members who responded to the survey in the given week)*100 | Time-trend analyses, data dashboard, weekly reports |
| Belief | First belief | Definition: binary (yes/no) variable where panel members who believed the information item was “definitely true”, “seems like it could be true” or “not sure if it’s true or untrue” the first time they reported they heard/read/seen the information item were coded as a yes. | Sub-group analyses |
|  | Weekly belief | Definition: percentage of panel members who believed the information item was “definitely true”, “seems like it could be true” or “not sure if it’s true or untrue” and reported that they heard/read/seen the information item in a given week.    (Panel members who reported believing the information item in the given week/panel members who reported being exposed to the information item in the given week)*100 | Time-trend analyses, data dashboard, weekly reports |
