# Supplementary material for: iHeard STL: Development and first year findings from a local surveillance and rapid response system for addressing COVID-19 and other health misinformation
Source: PLoS One. 2023 Nov 3;18(11):e0293288. doi: 10.1371/journal.pone.0293288 (PMC10624282; doi:10.1371/journal.pone.0293288)
Supplement: S2 Table — (DOCX) [file pone.0293288.s002.docx]

**Table S2.** Subcategories for open-ended misinformation survey responses.

| **Subcategory** |
| --- |
| side effects |
| effectiveness |
| quality |
| nursing homes |
| treatment |
| development |
| fatalism |
| booster |
| policy |
| breakthrough |
| previous infection |
| vaccine rate |
| safety |
| political agenda |
| influenza |
| statistics |
| falsification |
| long term |
| hospitalization |
| Ivermectin |
| young children |
| line cutting |
| J&J |
| Moderna |
| vaccine unnecessary |
| financial interest |
| workplace |
| mandate |
| Pfizer |
| Democratic Administration |
| vaccination status |
| masks |
| variants |
| blood clots |
| cases |
| deaths |
| international |
| pills |
| infusions |
| patch |
| testing |
| symptoms |
| immunity |
| government control |
| public figure |
| fertility |
| religion |
