# Supplementary material for: iHeard STL: Development and first year findings from a local surveillance and rapid response system for addressing COVID-19 and other health misinformation
Source: PLoS One. 2023 Nov 3;18(11):e0293288. doi: 10.1371/journal.pone.0293288 (PMC10624282; doi:10.1371/journal.pone.0293288)
Supplement: S3 Table — (DOCX) [file pone.0293288.s003.docx]

**Table S3.** Beta estimates for weekly exposure and weekly belief rate.

|  | **Exposure** | | **Belief** | |
| --- | --- | --- | --- | --- |
|  | **Beta estimate (95% CI)** | ***p*-value** | **Beta estimate (95% CI)** | ***p*-value** |
| VaxFail | **-3.33 (-5.35, -1.30)** | **.008** | 1.43 (-2.60, 5.46) | .404 |
| VaxDanger | **-2.67 (-3.88, -1.46)** | **.001** | 1.97 (-0.55, 4.49) | .106 |
| KidMask | **-8.11 (-10.22, -6.00)** | **.004** | -1.30 (-17.56, 14.97) | .764 |
| Ivermectin | **-2.63 (-4.03, -1.23)** | **.003** | -1.57 (-3.93, 0.79) | .160 |
