# Supplementary material for: iHeard STL: Development and first year findings from a local surveillance and rapid response system for addressing COVID-19 and other health misinformation
Source: PLoS One. 2023 Nov 3;18(11):e0293288. doi: 10.1371/journal.pone.0293288 (PMC10624282; doi:10.1371/journal.pone.0293288)
Supplement: S4 Table — (DOCX) [file pone.0293288.s004.docx]

**Table S4.** Cross tabulated counts of first exposure and first belief by subgroup.

|  | **First Exposure** | | | | | | | | **First Belief** | | | | | | | |
| --- | --- | --- | --- | --- | --- | --- | --- | --- | --- | --- | --- | --- | --- | --- | --- | --- |
|  | **VaxFail** | | **VaxDanger** | | **KidMask** | | **Ivermectin** | | **VaxFail** | | **VaxDanger** | | **KidMask** | | **Ivermectin** | |
|  | **Yes** | **Unsure/ No** | **Yes** | **Unsure/ No** | **Yes** | **Unsure/ No** | **Yes** | **Unsure/ No** | **True/**  **Unsure** | **Not True** | **True/**  **Unsure** | **Not True** | **True/**  **Unsure** | **Not True** | **True/**  **Unsure** | **Not True** |
| **Age** |  |  |  |  |  |  |  |  |  |  |  |  |  |  |  |  |
| 18-39 | 25 | 31 | 19 | 64 | 21 | 33 | 22 | 58 | 6 | 30 | 4 | 36 | 2 | 27 | 5 | 32 |
| 40-49 | 6 | 11 | 5 | 14 | 8 | 8 | 7 | 11 | 2 | 8 | 1 | 10 | 1 | 9 | 0 | 8 |
| 50 and older | 9 | 13 | 10 | 16 | 12 | 9 | 9 | 15 | 4 | 10 | 2 | 11 | 5 | 8 | 4 | 10 |
| **Sex** |  |  |  |  |  |  |  |  |  |  |  |  |  |  |  |  |
| Male | 12 | 10 | 10 | 18 | 10 | 12 | 10 | 18 | 2 | 11 | 1 | 13 | 3 | 8 | 3 | 9 |
| Female | 27 | 43 | 23 | 74 | 29 | 37 | 26 | 65 | 10 | 35 | 6 | 43 | 5 | 33 | 6 | 39 |
| **Race** |  |  |  |  |  |  |  |  |  |  |  |  |  |  |  |  |
| white | 25 | 40 | 16 | 69 | 29 | 35 | 29 | 53 | 4 | 36 | 1 | 40 | 3 | 34 | 4 | 38 |
| Black or African American | 13 | 13 | 15 | 19 | 9 | 14 | 8 | 23 | 7 | 10 | 6 | 14 | 3 | 9 | 5 | 9 |
| **Participant Type** |  |  |  |  |  |  |  |  |  |  |  |  |  |  |  |  |
| Community Member | 10 | 17 | 11 | 38 | 14 | 11 | 14 | 33 | 2 | 14 | 1 | 18 | 4 | 12 | 2 | 15 |
| Front-line Worker | 30 | 38 | 23 | 56 | 27 | 39 | 24 | 51 | 10 | 34 | 6 | 39 | 4 | 32 | 7 | 35 |
