# Supplementary material for: iHeard STL: Development and first year findings from a local surveillance and rapid response system for addressing COVID-19 and other health misinformation
Source: PLoS One. 2023 Nov 3;18(11):e0293288. doi: 10.1371/journal.pone.0293288 (PMC10624282; doi:10.1371/journal.pone.0293288)
Supplement: Attachment — Submitted filename: Response to Reviewers.docx [file pone.0293288.s006.docx]

**Response to Reviewers**

**Reviewer #1:** **General comments - This paper addresses an important topic of misinformation during pandemic times, when members of the public were exposed to high volumes of accurate information, misinformation, and disinformation. The study employed a public health surveillance model to assess prevalence of misinformation, and had high survey response rates. The study generated very interesting findings including the feasibility of a local misinformation surveillance system, and the persistence of misinformation beliefs over time despite routine counter-messaging.

1. Introduction
- Consider including a definition of “misinformation.”**

**Author response:** Thank you for this comment. We have added a definition in the first sentence of the introduction on line 49 of the tracked changes document.

**2. Consider including more information around COVID disparities to set the stage for why you specifically recruited a large proportion of Black community members to be a part of the panel.**

**Author response:** Thank you for the comment. We added a phrase to the introduction on lines 53 to 55 of the tracked changes document that sets the stage for our Black community focus.

**Methods**
**3. Panel composition. The authors describe their efforts to recruit a diverse panel with intentionality to include members of certain under-represented demographics including Black community members and community members from certain zip codes. They also sought representation of front-line workers in the sample and provide a definition they used for this term. It is worth noting that front-line workers encompassed healthcare workers – are these healthcare workers representative of the general population when it comes to receiving health information? Among the front-line workers, do the authors know what proportion were in healthcare?**

**Author response:** Our operational definition of frontline workers was comprised of individuals who engage with community members through in-person, online, or phone interactions on our recruitment survey. Individuals self-classified themselves according to this definition on the recruitment survey. This inclusive approach was intended to encompass a diverse range of professionals, such as healthcare workers, phone operators, and social service personnel. We appreciate your insightful feedback and have conducted additional data analyses in response, where two reviewers coded open-ended responses on occupations as healthcare workers or not. In our analyses, we took into account missing data for 6 of the 214 panelists on occupation and two conflicts where one reviewer was uncertain and the other thought they were non-healthcare workers. To accommodate these uncertainties, we estimated the percentage of healthcare workers under four different possible scenarios:

1. The two uncertain cases and all with missing data were non-healthcare workers,

2. The two uncertain cases were healthcare workers and all with missing data were non-healthcare workers,

3. The two uncertain cases were non-healthcare workers and all with missing data were healthcare workers,

4. The two uncertain cases and all with missing data were healthcare workers.

Our analysis showed that healthcare workers constituted an estimated 8.4 to 12.2% of our overall participant pool, and an estimated 15.3 to 22% of these individuals fell within the category of frontline workers. We have integrated this observation into our Discussion section to provide further transparency about our sample composition. Specifically, we added the text on lines 605 to 609 on the tracked changes version of the discussion section: “In addition, an estimated 8.4 to 12.2% of study population were employed in the healthcare sector (of which an estimated 15.3 to 22% were frontline workers) (data not shown) vs. 14% of the U.S. population employed in the healthcare sector [40]. This may cause underestimated exposure rates on surveyed health-related claims.”

**4. The authors should include information in the methods regarding timing of their surveys in relation to the pandemic. Was this conducted at a time of surge or low virus circulation? What were local public policies at the time? (I believe their surveys started around the time or shortly after the Delta surge in the summer of 2021).**

**Author response:** We added two sentences to the data collection paragraph on lines 137 to 140 of the tracked changes version to describe virus circulation during this time as well as local public policies on masking.

**5. The authors provide a thorough description of how beliefs and exposures were captured.**

**Author response:** We thank the reviewer for this comment.

**6. Were surveys provided in English only? If so, would this help explain the low participation of Hispanic community members? Language of surveys should be noted in methods, and potential biases should be noted in limitations.**

**Author response:** We thank the reviewer for this comment. We have added this information to the data collection section and to the last sentence of the limitations section of the discussion section on lines 618 to 626 of the tracked changes version.

**Discussion**
**7. For the paragraph starting in line 383, nuanced framing of discussion in differences between racial/ethnic groups is warranted. This is especially important given stark inequities in COVID-related health outcomes between these groups. Readers should not walk away from this paragraph thinking that members of the Black community are more “gullible” (ie likely to believe false information) due to their “culture.” This is somewhat implied as the paragraph currently stands, though I do not think this was the authors’ intent. This paragraph must include a more robust discussion, including explicit consideration of how structural factors including structural violence, institutional racism, and even interpersonal racism influence trust in biomedical health system and where community members access health information. It is very important to specifically call out racism and its impact on mistrust of the biomedical system. See for example:** [**https://www.ncbi.nlm.nih.gov/pmc/articles/PMC7241063/**](https://www.ncbi.nlm.nih.gov/pmc/articles/PMC7241063/)

**Author response:** We thank the reviewer for this important comment. We have made the following edits to this paragraph. We have dropped the sentence: “These findings are consistent with other recent studies that have highlighted how history, culture, and other socioecological factors influence belief of misinformation among different sociodemographic groups [33–35]” and replaced it with on lines 479 to 482 of the tracked changes version “There is a long history of African Americans being targeted with health harming products and information as well as mistrust of health care and health research among Africans Americans based on past abuses [30,31].” Further, we have replaced “higher odds of believing” with “higher odds of believing an item could be true or being uncertain whether it’s true or untrue” in the same paragraph.

**8. (Similarly, the framing of racial differences in the abstract must also be addressed. The current wording regarding differences in belief of misinformation between Black and white community members is not nuanced enough and does not adequately address the systemic factors. )**

**Author response:** We thank the reviewer for this comment. We have added a sentence at the end of the conclusion to highlight that further studies are needed to understand the observed racial differences in exposure and belief on lines 34 to 36 of the tracked changes version.

**9. The exclusion of members from certain demographics in the analysis due to small numbers may have biased the data. This should be noted in the limitations.**

**Author response:** We thank the reviewer for this comment. We added a sentence about this to the limitations section on lines 613 to 616 of the tracked changes version.

**10. The limitations can also address not knowing what proportion of front-line workers were in fact healthcare workers, and discussion of how this may have impacted the findings.**

**Author response:** We have addressed this in the discussion section as described in response 3.

**Conclusion**
**11. Lines 456-458 - Consider using updated terminology for monkeypox (i.e. Mpox), and also could frame this more broadly around other emerging diseases.**

**Author response:** We have changed the conclusion where noted on line 635 of the tracked changes version to include “(e.g., Mpox and other emerging diseases)”.

**Minor points –**
**12. Typically we do not capitalize the ‘w’ in white, unless otherwise instructed in journal stylebook.**

**Line 291 – typo “we not included”**

**Author response:** We thank the reviewer for this comment. We have made both of the changes as suggested.

**Reviewer #2:** **This manuscript describes the authors' experience with developing a surveillance and rapid response system to address COVID-19 misinformation in St. Louis, MI, community during the COVID pandemic.**

**The following issues have been identified following the review;**

**1. The ethical issues related to the study area are not adequately discussed. Specifically, there is a need for the authors to comment on the consent processes followed during the study.**

**Author response:** We have elaborated on the process in more detail on lines 119 to 129 of the panel member subsection in the methods in the tracked changes version. Specifically, we added the following text: “We shared recruitment materials with our partners and/or distributed them while at community-based events hosted by our partners. Individuals could access the recruitment form through scanning a QR code on the recruitment flyer or email the study email address that was provided on the recruitment material. The recruitment form includes eligibility questions, followed by a full description of the project and what is asked of participants if they volunteer to participate. For those who wish to participate after reading the informed consent information, they provide contact information for payment purposes and to receive the longitudinal survey.”

**2. The program iHeard STL from which the data emanate, has not been described adequately. There is need to provide more details regarding iHeard STL. e.g URL reference..what happened to it after the study? How is it maintained/operated?**

**Author response:** In response to the reviewer’s comment, we have updated the background to provide more information on *iHeard STL* on lines 90 to 99 of the methods section in the tracked changes version.

**3. There is need to comment on the sample size used for the analysis. How was this estimated? How was the sampling done (other than intentionally recruiting from the study area and targeting specified demographic characteristics).**

**Author response:** We primarily recruited through the partners listed in the methods who communicated about our study to members in their networks. Importantly, *iHeard STL* emerged as a local response to misinformation, not a planned research project. As with many aspects of the public health response to COVID-19, our panel size was determined by urgency and practicality – how many panel members could we enlist quickly, and how many could we afford to pay? It was not estimated for scientific purposes. We have incorporated the additional information regarding the context for its origin and sample size on lines 94 to 99 of the Methods background subsection in the tracked changes version.

**4. Table 3 does not show all columns. The columns on the right side are not complete.**

**Author response:** We apologize for not catching this during submission. This has now been corrected.

**5. The present study limited analyses to univariable analysis and did not adjust for confounding factors unlike the studies used for comparison in the discussions. Unlike the present study, these studies adjusted for confounders. It is not clear why the authors did not conduct multivariable regression analyses to account for confounders. This is major limitation that limits the validity of the conclusion from this analysis.**

**Author response:** We thank the reviewer for this comment. Our primary reason for not running a multivariable regression model was the limited sample size. We now acknowledge this sample size limitation in the strengths and limitations section on lines 617 to 618 in the tracked changes version.

**6. Although the finding on VaxDanger by race was statistically significant, it also has a very wide confidence interval. The authors need to expand the discussion relative to this finding.**

**Author response:** We thank the reviewer for this comment. The wide confidence interval is because of the small sample size for this analysis. We mention this in the limitation section and ask the readers to interpret it with caution. In addition, we performed Fisher’s exact test on each of the first-time exposure and first-time belief results, and the significance remained the same.

**7. For the study, participants were incentivized to complete the survey. What measures are in place to ensure the high level of engagement reported in the study is maintained when participants are no longer incentivized?**

**Author response:** We plan to continue to provide incentives for as long as there is funding for this surveillance system.
